# 用以监测和预测癌症疗效的分子诊断

**DOI:** 10.3779/j.issn.1009-3419.2011.07.15

**Published:** 2011-07-20

**Authors:** William CS CHO, 娟 南

**Affiliations:** 1 Molecular Diagnostics for Monitoring and Predicting Therapeutic Effect in Cancer; 2 天津医科大学总医院，天津市肺癌研究所，天津市肺癌转移与肿瘤微环境重点实验室; 3 香港特别行政区 伊利沙伯医院 临床肿瘤科

**Keywords:** 癌症生物标记物, 循环肿瘤细胞, 分子诊断, 预测标记物, 治疗反应

**“……许多研究证实，研发分子诊断生物标记物来监测和预测各种癌症的疗效是切实有效的……未来的研究重点是促使标记物从基础研究走向临床应用。”**

癌症研究增进了我们对癌症生物学的认识，这促使了癌症的新型诊断方法和有效治疗措施的研发^[1]^。多数癌症治疗方法仅对接受治疗的部分患者有效，因此，亟待通过加强分子诊断来改善癌症患者的预后，分子标记物在实现这一转变中具有重要作用^[2]^。本文将特别强调一些促进新型诊断方法和干预策略发展的癌症研究。通过重点介绍最新分子诊断癌症研究的发现，为充分认识用以监测和预测癌症疗效的分子标记物提供深刻的见解。

**“有助于预测疗效的标记物的鉴定是癌症预后研究的重要领域。”**

## 用以早期癌症诊断的分子生物标记物

能鉴别出对治疗具良好反应的早期癌症患者将减少该疾病的死亡率。在黑色素瘤进展的过程中测定患者血液中脂肪酸结合蛋白-7（fatty acid-binding protein-7, *FABP7*）的表达，结果发现，*FABP7*t^+^循环肿瘤细胞（circulating tumor cells, CTCs）可减少疾病的进展。这一肿瘤进展基因有可能成为早期黑色素瘤患者血液中的潜在诊断标记物^[3]^。

## 对放疗效果具有预测作用的分子标记物

人和鼠肺癌细胞的研究均显示，在暴露于电离辐射的最初数小时内及在治疗相关细胞凋亡和死亡出现之前，细胞内TIP-1（Tax-interacting protein 1）会移位至质膜表面。因此，辐射诱导的TIP-1移位至癌细胞表面的成像可以及时预测癌症对辐射的反应^[4]^。另一项研究发现，候选干细胞标记物CD44的mRNA和蛋白水平均与早期喉癌患者对放疗的反应显著相关。他们的结果提示，CD44的表达可预测喉癌放疗后的局部复发^[5]^。

## 对治疗药物的疗效具有预测作用的分子标记物

有助于预测疗效的标记物的鉴定是癌症预后研究的重要领域。两项早期乳腺癌的研究报道，辅助化疗后血液中人细胞角蛋白19（cytokeratin- 19, *CK-19*) mRNA阳性CTCs的检出是化疗耐受残留病存在的独立危险因素，*CK-19*能否成为监测微小残留病的标记物值得进一步的研究^[6, 7]^。在一项抗血管生成疗法治疗晚期甲状腺癌的Ⅱ期研究中，发现晚期甲状腺癌患者开始治疗后血清*PlGF*和可溶性*VEGF*受体2水平的改变可预测肿瘤对莫替沙尼（motesanib）的反应。*VEGF*的基线水平越低，患者的无进展生存期则越长^[8]^。

一些新近研究重点关注了多标记物检测的研发。在一项有关生物化疗（biochemotherapy, BCT）联合维持生物治疗的多中心Ⅱ期临床试验中，诱导BCT期间连续监测5种黑色素瘤相关CTC生物标记物（*MART-1*、*GalNAc-T*、*PAX-3*、*MAGE-A3*和*Mitf*）有助于预测接受BCT和维持生物治疗的Ⅳ期黑色素瘤患者的治疗效果和疾病预后^[9]^。另一项研究在确诊Ph染色体阳性急性淋巴母细胞白血病（Philadelphia chromosome-positive acute lymphoblastic leukemia）的成年患者骨髓样本中鉴定出了基于通路的生物标记物，可预测患者对酪氨酸激酶抑制剂（tyrosine kinase inhibitors, TKIs）联合化疗的治疗反应。他们利用9个基因标记可区分治疗反应良好的患者与伴有持续残留病和早期复发的患者^[10]^。在一项有关乳腺癌的多中心随机试验中，一种预测病理完全缓解的30个基因标记可识别对术前紫杉醇、氟脲嘧啶、阿霉素和环磷酰胺化疗敏感性较高的患者^[11]^。

miRNAs在肿瘤形成中发挥重要作用，它们对近年的癌症研究产生了革命性的影响^[12]^。一些研究报道，患者miRNA表达情况亦与癌症治疗反应相关^[13]^。一项研究显示，肝脏特异miRNA *miR-122*水平较低的慢性丙肝肝癌患者对干扰素治疗的反应较差^[14]^。另一项研究发现，在肝癌患者中，与*miR-26*表达水平较高的肿瘤患者相比，*miR-26*表达水平较低者对IFN-α辅助治疗的反应较好^[15]^。

另一方面，一项前瞻性研究评估了^18^F-FDG PET/CT在预测患者对新辅助化疗EGF受体（EGFR）-TKI反应中的作用。结果发现，在厄洛替尼治疗疗程的早期，^18^F-FDG PET/CT可预测非小细胞肺癌患者对术前厄洛替尼治疗的反应^[16]^。

## 对癌症患者的转移情况和生存期具有预测作用的分子标记物

预后良好或不良的患者的鉴别对有效治疗方案的研发至关重要。在此重点介绍一些致力于发现预后生物标记物的新近研究。

有一项研究比较了早期雌激素受体阳性乳腺癌女性患者接受术前短期他莫昔芬治疗后的Ki-67标记指数，结果发现，药物治疗后Ki-67≥20%的患者的死亡风险较 < 20%的患者高5.5倍^[17]^。另一项研究检测了采用辅助铂类化疗的原发性卵巢癌患者的氧化还原蛋白表达，结果发现，肿瘤中细胞质低表达而细胞核高表达硫氧还蛋白的患者的无进展生存期和总生存期较长^[18]^。

**“miRNAs在肿瘤形成中发挥重要作用，它们对近年的癌症研究产生了革命性的影响”**

一项欧洲癌症研究与治疗组织（European Organisation for Research and Treatment of Cancer, EORTC）的次要研究18991报道，通过定量实时逆转录聚合酶链反应（quantitative real-time reverse transcription- PCR, qRTPCR）检出外周血CTC的酪氨酸酶和*MART-1*转录因子是Ⅲ期黑色素瘤患者无远处转移生存期的时间依赖性预后因素^[19]^。一项类似的研究定量评估了前哨淋巴结中黑色素细胞分化抗原和血管生成生物标记物，他们发现，酪氨酸酶、*MART-1*、*VEGF*和*PAI1*的表达与微转移显著相关。组织学结果呈阴性和酪氨酸酶的表达超过TATA盒结合蛋白的27个拷贝与复发或死亡风险增加显著相关^[20]^。有趣的是，一项新近研究显示，在不表达*MART-1* mRNA的转移性促纤维组织增生性黑色素瘤中检测到高分子量-黑色素瘤相关抗原（high-molecular- weightmelanoma- associated antigen, *HMW-MAA*）mRNA，提示通过qRT-PCR联合分析*HMW-MAA*和*MART-1*可提高mRNA评估可能转移至淋巴结的高危促纤维组织增生性黑色素瘤的敏感性^[21]^。

在一项研究局部晚期膀胱癌患者的福尔马林固定石蜡包埋（formalin-fixed paraffin-embedded, FFPE）肿瘤样本的Ⅲ期试验中，多药耐药基因（multidrug resistance gene 1, *MDR1*）和切除修复交叉互补基因1（excision repair crosscomplementing 1, *ERCC1*）基因的高表达与铂类辅助化疗效果不良相关^[22]^。一项前瞻性多中心试验分析结直肠癌患者石蜡包埋的前哨淋巴结和血液标本，发现通过qRTPCR方法检测4种分子标记物（*c-MET*、黑色素瘤抗原A3基因家族、β1→4-*N*乙酰半乳糖胺基转移酶和细胞角蛋白20）在监测转移中起重要作用，并与总生存期相关^[23]^。癌症药物研发的未来为靶向作用于各种通路而非单个基因，因此，基于通路的分子标记物在抗击癌症的战斗中将发挥关键作用。一个研究小组在4种功能性通路（细胞周期、凋亡、巨噬细胞活化和干扰素调节因子4）的基础上建立了一个11个基因危险评分预测模型来研究晚期典型霍奇金氏淋巴瘤的FFPE样本，以辨别5年无失败生存率不同的低危和高危患者^[24]^。

另一方面，在肺癌中，miRNAs也被认为是有前景的预后标记物^[25, 26]^。有报道称，*miR-34a*的低表达与非小细胞肺癌手术切除后的高复发率相关。伴有*p53*突变和*miR-34a*低表达的患者的复发率最高^[27]^。

## 新兴和新型的分子诊断

CTC作为癌症患者治疗反应和预后的有效替代标记物具临床意义。尽管近年来CTCs的检测已取得一定进展，但仍亟待我们发现更好的技术来提高检测实验的敏感性和可靠性。高通量组学技术的开发有助于更深入地阐释癌症，为改变癌症诊断的现状提供了分子知识基础^[28]^。

**“最终目标是研发临床诊断方法以便医生针对个体化癌症患者作出最佳诊断。”**

一项研究采用膜滤过分离肿瘤细胞技术（isolation by size of epithelial tumor cell, ISET）来检测皮肤黑色素瘤患者的CTCs，结果发现，在原位黑色素瘤患者中未检测到CTCs，而在原发性侵袭或转移的黑色素瘤患者中可见。结果显示，通过ISET检测黑色素瘤患者外周血中的CTCs可提供转移进程的相关信息，有可能促进患者的及时治疗^[29]^。

由于缺乏直接作用于表面细胞抗原的良好单克隆抗体来捕获癌细胞，通过qRT-PCR分析来评估血液中CTCs较为困难。最近研发了一种*HMW-MAA*特异性单克隆抗体偶联免疫磁珠联合PCR分析，以评估黑色素瘤患者的CTCs。这一分析可检测Ⅳ期黑色素瘤患者的*MLANA*、*MAGEA3*和*MITF*的表达，以及*BRAFmt*（一种编码V600E突变蛋白的*BRAF*基因变异）的存在^[30]^。

一项采用表达绿色荧光蛋白（green fluorescent protein, GFP）的端粒酶特异的选择性增殖腺病毒的新技术已建立，以期在成千上万的的外周血白细胞中肉眼观察到活的人CTCs。该检测方法涉及3个步骤，包括红血细胞溶解，随后向细胞沉淀中加入表达GFP的减毒腺病毒以及采用荧光显微镜自动扫描^[31]^。GFP可监测活细胞过程的性能，使得科学家可研究完整有机体的细胞过程和发育过程的动力学。在不久的将来，我们无疑会看到GFP更多地用作癌症基因表达的标记物。

## 分子诊断在癌症治疗中的意义及前景

在过去的几年中，许多研究证实，研发分子诊断标记物来监测和预测各种癌症的疗效是切实有效的^[2]^。2007年EORTC 10994/乳腺国际组织（Breast International Group, BIG）00–01临床试验的子研究报道，他们已获得并验证了可预测乳腺癌对紫杉醇辅助化疗的反应的特异性标记物^[32]^。该项结果对后续临床试验意义重大。但是，一些新的研究结果使得人们对这预测治疗反应的方法产生忧虑。导致3项在研临床试验最近被暂停，以待对这些临床试验和这用作治疗决策的预测模型作详尽的独立调查^[33]^。这一事件揭示了生物标记物开发和验证的重要性。

最近的技术进展促使发现了许多用以分子诊断的肿瘤标记物。过去的教训提醒我们，建立生物标记物分析不宜仅关注敏感性和特异性。尤其当分子标记物用以制定治疗策略时，应考虑目标人群的临床有效性。未来的研究重点是促使生物标记物从基础研究走向临床应用。最终目标是研发临床诊断方法以便医生针对个体化癌症患者作出最佳诊断。

## Financial & competing interests disclosure

The author has no relevant affiliations or financial involvement with any organization or entity with a financial interest in or financial conflict with the subject matter or materials discussed in the manuscript. This includes employment, consultancies, honoraria, stock ownership or options, expert testimony, grants or patients received or pending, or royalties.

No writing assistance was utilized in the production of this manuscript.
